# Potential use of local and systemic humoral immune response parameters to forecast *Mycoplasma hyopneumoniae* associated lung lesions

**DOI:** 10.1371/journal.pone.0175034

**Published:** 2017-04-05

**Authors:** Beatriz Garcia-Morante, Joaquim Segalés, Lorenzo Fraile, Gemma Llardén, Teresa Coll, Marina Sibila

**Affiliations:** 1 IRTA, Centre de Recerca en Sanitat Animal (CReSA, IRTA-UAB), Campus de la Universitat Autònoma de Barcelona, Bellaterra, Spain; 2 Boehringer Ingelheim España S.A., Sant Cugat del Vallès, Spain; 3 UAB, Centre de Recerca en Sanitat Animal (CReSA, IRTA-UAB), Campus de la Universitat Autònoma de Barcelona, Bellaterra, Spain; 4 Departament de Sanitat i Anatomia Animals, Facultat de Veterinària, Campus de la Universitat Autònoma de Barcelona, Bellaterra, Spain; 5 Departament de Ciència Animal, ETSEA, Universitat de Lleida, Lleida, Spain; 6 Boehringer Ingelheim Veterinary Research Center GmbH&Co., Hannover, Germany; Universidade Federal de Pelotas, BRAZIL

## Abstract

Immunopathological events are key for the development of enzootic pneumonia (EP), which is macroscopically observed as cranioventral pulmonary consolidation (CVPC). This study aimed to investigate the putative association between the humoral immune response against *Mycoplasma hyopneumoniae* (*M*. *hyopneumoniae*) and prevalence and extension of CVPC in 1) experimentally infected pigs, 2) slaughtered pigs and 3) sequentially necropsied pigs in a longitudinal study. CVPC was scored by means of the European Pharmacopoeia recommended methodology. Specific IgG, IgG1 and IgG2 antibodies were assessed in serum. In addition, mucosal IgG and IgA antibodies were analyzed in broncho-alveolar lavage fluid (BALF) from experimentally challenged pigs. The systemic humoral immune response in experimentally infected pigs was delayed in onset whereas humoral respiratory mucosal immune response appeared more rapidly but declined earlier. Although low, BALF IgG antibodies showed the highest correlation with CVPC scores (r = 0.49, *p*<0.05). In slaughter-aged pigs, both percentage of lungs with CVPC and mean lung lesion score were significantly higher in *M*. *hyopneumoniae* seropositive farms compared to the seronegative ones (*p*<0.001). Similarly, seropositive sequentially necropsied pigs showed more severe CVPC than seronegative ones. Overall, mean serological values might help to forecast prevalence and severity of EP-like lung lesions using a population based approach. Remarkably, the specific systemic humoral immune response was found to be predominated by the IgG2 subclass, suggesting a dominant Th1-mediated immune response to *M*. *hyopneumoniae*.

## Introduction

One of the main pulmonary lesions found in pigs on abattoir inspection is cranioventral pulmonary consolidation (CVPC) [1, 2]. *Mycoplasma hyopneumoniae* (*M*. *hyopneumoniae*) is considered the most important primary bacterial respiratory pathogen involved in such lung lesions. When secondary or concomitant infections by other respiratory bacterial organisms are also implicated, enzootic pneumonia (EP) occurs [3].

CVPC quantification by means of lung lesion scoring is frequently used to estimate the incidence and severity of lung lesions associated to *M*. *hyopneumoniae* infections, at experimental, herd and abattoir levels [4–6]. Nevertheless, *post-mortem* lung evaluation is an end-point parameter that does not provide information on the ongoing respiratory problems in any of the cases [6]. Thus, it would be of general interest to find an *ante-mortem* parameter that would provide reliable and real-time information in relation with CVPC prevalence and severity.

Immunopathological events are considered to play an important role in both *M*. *hyopneumoniae* infection pattern and development of the associated lung lesions [3]. In connection with this matter, relationship between presence of *M*. *hyopneumoniae* antibodies and CVPC development has been analyzed in several studies. However, while some studies found a significant positive correlation between both parameters [2, 7–10], others did not find such association [1, 11]. Noteworthy, such relationship has been assessed at a population-based level and scarce information is available at individual level [10]. Hence, the relationship between antibody levels against *M*. *hyopneumoniae* as a tool to assess lung lesions deserves further investigation. On the other hand, most of the commercially available enzyme-linked immunosorbent assay (ELISA) kits are designed to detect specific *M*. *hyopneumoniae* IgG antibodies in serum, but information on other humoral immune parameters and their relationship with CVPC is not available. Therefore, the present study sought to investigate the potential association between different humoral immune parameters, both at local and systemic levels, with the prevalence and severity of CVPC in pigs naturally and experimentally infected with *M*. *hyopneumoniae*.

## Materials and methods

### Study design

**Fig 1:**
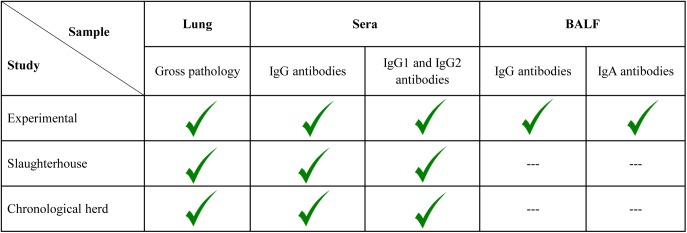
Parameters and samples evaluated within the experimental, slaughterhouse and chronological herd studies. Macroscopic lung lesions were quantified by the European Pharmacopoeia (Ph. Eur., monograph number 04/2013:2448) lung scoring system. All humoral immune parameters were assessed by ELISA technique.

In order to accomplish the abovementioned objective, samples from three different published studies were used: 1) samples from *M*. *hyopneumoniae* experimentally inoculated animals [4]; 2) samples from slaughtered pigs coming from different farms [2] and 3) samples from non-vaccinated pigs belonging to a field study in which chronological necropsies were performed [12]. Samples were tested to evaluate the humoral immune parameters schematized in Fig 1 and detailed further below.

#### Experimental study

Ninety seven 6 week-old pigs free from *M*. *hyopneumoniae* were experimentally inoculated as described previously [4]. One uninfected group (Control; n = 6) received sterile phosphate buffered saline (PBS). At 21 (n = 37) or 28 (n = 60) days post-inoculation (dpi), all pigs were killed with an intravenous overdose of sodium pentobarbital and subjected to necropsy examination. At that time (21 or 28 dpi), blood and broncho-alveolar lavage fluid (BALF) samples were obtained and CVPC evaluation was assessed using the system recommended by the European Pharmacopoeia (Ph. Eur., monograph number 04/2013:2448). Sera were used for specific IgG, IgG1 and IgG2 detection, whereas IgG and IgA antibodies were determined in BALF. Animal care and study procedures were conducted in accordance with the guidelines of Good Experimental Practice, under the approval of the Ethical and Animal Welfare Committee of the Universitat Autònoma of Barcelona.

#### Slaughterhouse study

A total of 54 batches of 54 different farms located in North-eastern, Central and South-eastern Spain, were included in the study [2]. A batch was defined as a group of pigs (with a mean of 97 animals per batch) belonging to the same farm and sacrificed on the same day at the slaughterhouse. From these 54 farms, 9 farms vaccinated pigs against *M*. *hyopneumoniae*, 16 did not vaccinate and, from the remaining farms, no data was available [2]. The pigs were killed in eight different slaughterhouses according to their own procedures. Percentage of lungs affected by CVPC as well as mean lung lesion score per farm (sum of individual scores/number of scored lungs) were assessed. In addition, blood samples from 20 randomly selected pigs of each batch were taken before being sacrificed and IgG, IgG1 and IgG2 *M*. *hyopneumoniae* specific antibodies were determined from sera. Farms were then classified as seropositive or seronegative according to the mean S/P ratio obtained from those 20 sampled animals. In addition, mean farm IgG1 and IgG2 OD values were also obtained.

#### Chronological herd study

Fifty-eight pigs from a farrow-to-finish farm located in North-eastern Spain with a history of EP associated respiratory problems were included in the analyses. These piglets belonged to the control group (non-vaccinated) of a previously published work [12]. Blood samples from these animals were taken at 1, 3, 6, 9, 12, 15, 18 and 22 weeks of age. However, the initial number of pigs decreased over time because animals were randomly sacrificed with an intravenous overdose of sodium pentobarbital, necropsied and CVPC evaluated from 9 weeks of age (n = 10) onwards at 12 (n = 12), 15 (n = 7), 18 (n = 13) and 22 (n = 16) weeks of age. Specific IgG antibodies were tested from all serum samples in each time point whereas IgG1 and IgG2 specific isotypes were assessed only from sera obtained at the necropsy time, from 9 to 22 weeks of age. Housing, husbandry and slaughtering conditions conformed to the European Union Guidelines and Good Clinical Practices, under the approval of the Ethical and Animal Welfare Committee of the Universitat Autònoma of Barcelona.

### Pathological examination

In each of these studies, extension of gross lung lesions compatible with *M*. *hyopneumoniae* infection (CVPC) was assessed using different scoring systems depending on the study. Thus, in order to harmonize all lung lesion scores, those that were obtained through Madec and Kobisch (1982) [13] (slaughterhouse study) and Hannan et al. (1982) [14] (chronological study) methodologies were converted to reference European Pharmacopoeia (Ph. Eur., monograph number 04/2013:2448) scores by means of the equivalence formulae recently provided [4].

### *M*. *hyopneumoniae* IgG antibody detection

Sera derived from all the studies were tested for *M*. *hyopneumoniae* IgG antibodies by means of a commercial indirect ELISA (*Mycoplasma hyopneumoniae* Antibody Test Kit; BioChek, UK) and according to the manufacturer’s instructions. The relative amounts of specific IgG antibodies in samples were expressed as sample-to-positive (S/P) ratio based on optical densities (OD) as follows: S/P = OD sample-OD mean negative control/ OD mean positive control-OD mean negative control. Samples with an S/P of ≥0.5 were considered positive whereas samples with an S/P of <0.5 were considered negative. An S/P = 0 was given to those samples with OD values below the mean OD of the negative control.

### *M*. *hyopneumoniae* IgG antibody subtype detection

*M*. *hyopneumoniae*-specific IgG1 and IgG2 subtypes from sera were measured by using a modification of the above mentioned ELISA (*Mycoplasma hyopneumoniae* Antibody Test Kit; BioChek, UK). Mouse anti-pig IgG1 and IgG2 monoclonal antibodies (Bio-Rad Laboratories, USA) were firstly tested in *M*. *hyopneumoniae* positive and negative sera diluted 1:30, 1:50, 1:70, 1:90, 1:120 and 1:150 with the sample diluent provided by the kit. As a result, 1:50 dilution (applied for total IgG antibodies determination according to manufacturer’s instructions) was appropriate to discriminate between *M*. *hyopneumoniae* seropositive and seronegative samples. After 30 min incubation, plates were washed and IgG1 and IgG2 antibodies diluted to 1:1000 were added. After incubating for 45 min, plates were washed again and alkaline phosphatase-labeled goat anti-mouse IgG polyclonal antibody (Bio-Rad Laboratories, USA) diluted to 1:1000 was added to each plate well. After incubating for 45 min, plates were washed and the reaction was visualized after 15 min incubation with the kit substrate reagent. Sample absorbance was obtained by reading at 405 nm and results expressed as OD values. For each plate, sample results were adjusted by subtracting the OD of the mean negative control provided by the kit. Furthermore, the cut-off was calculated as mean OD value of negative control plus three standard deviations (SD). The threshold was established at an OD value of 0.13 for both IgG1 and IgG2 antibodies.Values higher than this cut-off were considered positive whereas values below this cut-off were considered negative.

### Mucosal respiratory *M*. *hyopneumoniae* IgG and IgA antibody detection

Undiluted and previously centrifuged BALF samples from the experimental study were tested for specific IgG antibodies by means of the above mentioned assay (*Mycoplasma hyopneumoniae* Antibody Test Kit; BioChek, UK) and conforming to the manufacturer’s instructions but for the initial dilution of the sample. For IgA antibody detection, an alkaline phosphatase-labelled goat anti-porcine IgA polyclonal antibody (Bethyl Laboratories, USA) was used as described previously [15]. Sample absorbance was obtained by reading at 405 nm and results expressed as OD values after subtraction of the mean OD value of BALF from the negative control animals. The cut-off was calculated as mean OD value of the negative control plus three standard deviations (SD) for both IgG and IgA BALF antibodies and established at an OD value of 0.34 and 0.29, respectively. Whereas values higher than this cut-off were considered positive, values below this cut-off were considered negative.

### Statistical analyses

Statistical analyses were performed using SPSS software, version 15.0 (SPSS Inc., Chicago, Illinois, USA). The significance level (α) was set at *p*<0.05. Variables included in the statistical analyses were classified as categorical (presence or absence of CVPC and positive or negative S/P ratios and OD values for IgG1 and IgG2 in serum and for IgG and IgA in BALF) or continuous (S/P ratio, and IgG1 and IgG2 OD values in sera and IgG and IgA OD values in BALF, percentage of lungs showing CVPC and Ph. Eur. scores). A non-parametric test (Mann-Whitney) was used to evaluate the effect of any categorical variable over the continuous ones. Besides, in order to study putative associations among continuous variables, a linear regression analysis was carried out. Contingency tables with the corresponding chi-square statistics were performed among categorical variables.

## Results

### Experimental study

None of the control pigs showed CVPC. Among inoculated animals, local and systemic *M*. *hyopneumoniae* humoral immune parameters, percentage of animals showing CVPC as well as the mean lung lesion score of the animals sacrificed at 21 or 28 dpi are represented in Table 1.

**Table 1:** Immunopathological data obtained from the experimental study. **Number (%) of animals showing CVPC, mean Ph. Eur. Score (± SD), mean S/P ratio (± SD) and number (%) of seropositive (IgG) and positive animals to *M*. *hyopneumoniae*-IgG1 and IgG2 in sera and to IgG and IgA antibodies in BALF at 21 and 28 dpi.** Different superscripts within a column indicate significant differences among days post-inoculation (*p*<0.05).

	Lung pathology		Serology		Sera isotypes detection		BALF antibodies detection	
Days post-inoculation (dpi)	No. of animals with CVPC (%)	Mean Ph. Eur. Score (± SD)	No. of seropositive animals (%)	Mean S/P ratio (± SD)	No. of IgG1 positive animals (%)	No. of IgG2 positive animals (%)	No. IgG positive animals (%)	No. of IgA positive animals (%)
21 (n = 37)	30 (81.1)^a^	7.7 (± 8.1)^a^	3 (8.1)^a^	0.09 (± 0.22)^a^	0 (0)^a^	0 (0)^a^	34 (91.9)^a^	36 (97.3)^a^
28 (n = 60)	48 (80)^a^	9.5 (± 10.3)^a^	12 (20)^a^	0.26 (± 0.34)^b^	18 (30)^b^	32 (53)^b^	37 (61.7)^b^	45 (75)^b^

#### Lung lesion evaluation

CVPC was observed both in pigs sacrificed at 21 and 28 dpi and no significant differences (*p*>0.05) were found in terms of number of animals affected neither in mean lung lesion scores (Table 1).

#### Serology

Number of seropositive animals was higher at 28 than at 21 dpi (*p* = 0.09) (Table 1). Although below the positivity threshold, mean S/P ratio at 28 dpi was significantly higher than at 21. While all seropositive pigs (n = 15) showed CVPC with different levels of severity (from 0.4 to 32% of lung weight damaged), a total of 63 out of 82 seronegative animals (76.8%) had CVPC. In addition, no significant (*p*>0.05) correlation was found between individual lung lesion scores and S/P ratios. Nevertheless, the mean S/P ratio of animals with CVPC (0.32±0.36) was significantly higher (*p*<0.05) than the one from animals without lung lesions (0.03±0.05) at 28 dpi.

#### Serum IgG1 and IgG2 subclasses

**Fig 2:**
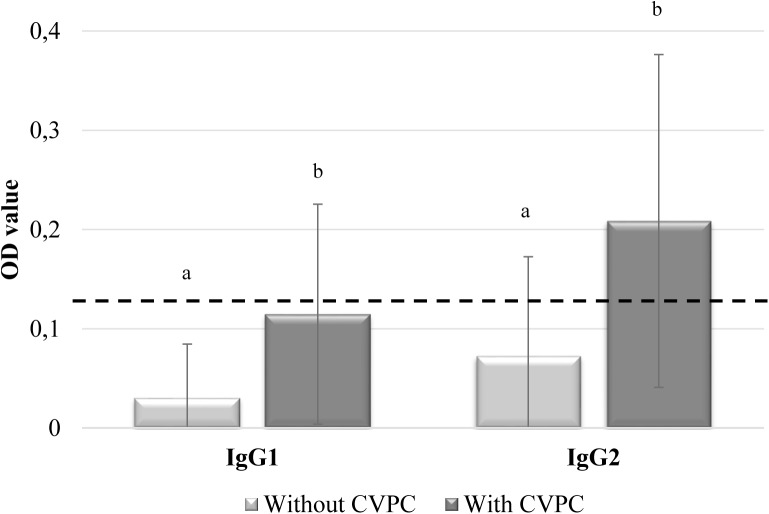
Mean IgG1 and IgG2 isotypes OD values (±SD) in animals with and without CVPC at 28 dpi from the experimental study. Different superscripts indicate significant differences of IgG1 and IgG2 antibody levels among animals with and without CVPC (*p*<0.05). Discontinuous line represents the positivity threshold.

Neither IgG1 nor IgG2 against *M*. *hyopneumoniae* were detected in sera at 21 dpi, even in those three animals that seroconverted for total IgG (Table 1). However, all the animals that seroconverted by 28 dpi (n = 12) were positive to both isotypes. A significant positive correlation (r = 0.77, *p*<0.0001) between OD values of both antibody subclasses was observed, but serum IgG2 amounts were found to be significantly higher (*p*<0.05) than IgG1 ones at 28 dpi. Furthermore, significantly higher (*p*<0.05) IgG1 and IgG2 levels were detected in those animals showing CVPC in comparison with those without lung lesions (Fig 2). However, no association between IgG1 or IgG2 OD values and lung lesion scores at individual animal level was observed.

#### Local humoral immune response

**Fig 3:**
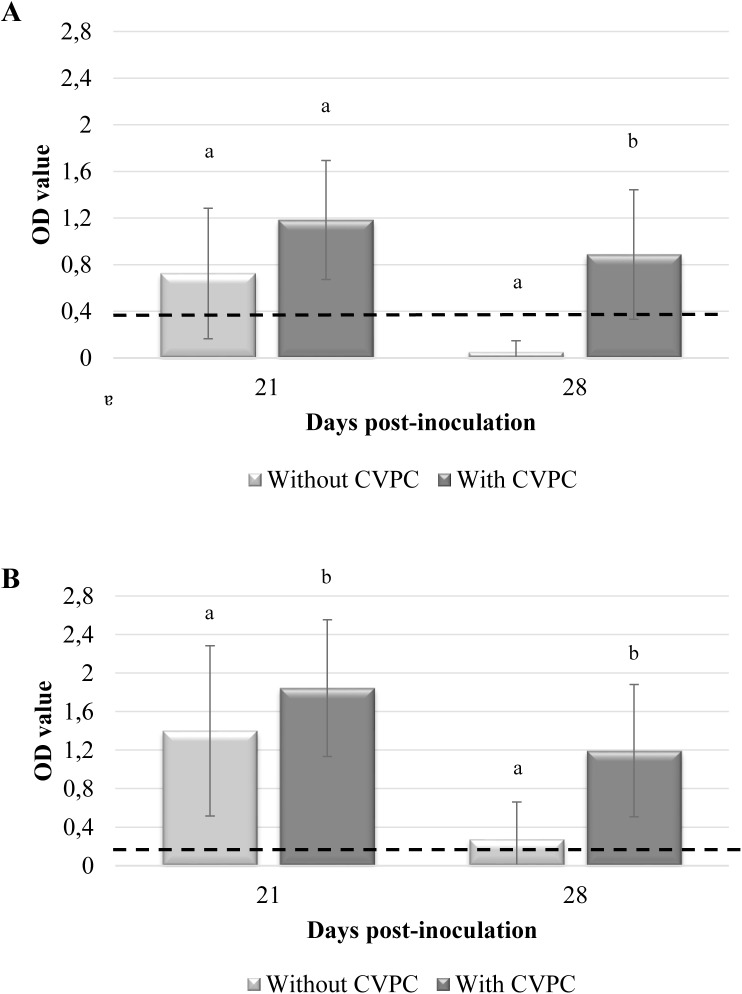
**Mean *M*. *hyopneumoniae* IgG (A) and IgA (B) OD values (±SD) in BALF samples from animals of the experimental study with and without CVPC at 21 and 28 dpi.** Different superscripts indicate significant differences of antibody levels among animals with and without CVPC at 21 and 28 dpi (*p*<0.05). Discontinuous line represents the positivity threshold.

Number of positive animals for BALF IgG and IgA antibodies was significantly higher (*p*<0.05) at 21 dpi than at 28 dpi (Table 1). A significant increase (*p*<0.05) of both specific IgG and IgA antibodies in BALF was observed in those animals showing CVPC in comparison with those without lung lesions (Fig 3A and 3B, respectively), but for specific IgG BALF antibodies at 21 dpi (*p* = 0.07) (Fig 3A). Although moderate to low, a significant positive correlation (r = 0.49, *p*<0.05) between IgG BALF OD values and lung lesion scores at individual level was observed. Nonetheless, this relationship was not observed with regard to IgA BALF OD values.

### Slaughterhouse study

#### Gross pathology and serology

All the 54 farms included in the present study were found to be affected by CVPC. Globally, mean percentage of lungs affected by CVPC and mean lung lesion score (Ph. Eur.) were 68.1 (min 34, max 99) and 5.15 (min 0.19, max 16.38), respectively. Based on the results obtained from the 20 randomly tested serum samples per farm, 40 (74.1%) farms were considered seropositive and the remaining 14 (25.9%) seronegative. Additionally, seropositive farms had a significantly higher (*p*<0.001) mean lung lesion score as well as percentage of lungs affected by CVPC than seronegative ones (Table 2). However, no significant relationship (*p*>0.05) was found between the mean S/P value of the 20 randomly collected sera and the mean lung lesion score at individual farm level.

**Table 2:** Mean Ph. Eur. Score (±SD) and percentage of affected lungs by CVPC (±SD) in seropositive and seronegative farms from the slaughterhouse study. Different superscripts within a row indicate significant differences among types of farms (*p*<0.05).

	Seropositive Farms	Seronegative Farms
**Mean Ph. Eur. Score (±SD)**	6.09 (± 3.89)^a^	2.47 (± 1.91)^b^
**Mean percentage of affected lungs (±SD)**	71.73 (± 12.78)^a^	57.57 (± 12.07)^b^

#### Serum IgG1 and IgG2 subclasses

All the 40 farms classified as seropositive showed positive IgG2 humoral immune responses and 24 of them (60%) had also IgG1 positive reactions. Similarly to what was observed at experimental level, a significant positive correlation (r = 0.59, *p*<0.0001) between both isotypes was detected and IgG2 response against *M*. *hyopneumoniae* was found to be significantly (*p*<0.0001) higher than the IgG1 one. Indeed, a significant positive correlation was found between S/P ratios and IgG2 OD values (r = 0.83, *p*<0.0001) whereas such relationship was not as good when considering IgG1 OD values (r = 0.48, *p* = 0.0002). Though, no significant association (*p*>0.05) between the mean IgG2 OD value and the mean lung lesion score at individual farm level was found.

### Chronological herd study

#### Gross pathology and serology

*M*. *hyopneumoniae* serological results from all monitored pigs and pathological results obtained along sequential necropsies are summarized in Table 3. From 1 to 12 weeks of age, the percentage of seropositive pigs was below 10%. However, from 15 weeks of age onwards, percentage of seropositive pigs increased progressively towards the end of the finishing period. Animals with CVPC began to appear from 12 weeks of age onwards. By the end of the study (22 weeks), the highest percentage of seropositive animals paralleled with the highest mean S/P values. Although not statistically significant (*p*>0.05), seropositive animals necropsied at 15, 18 and 22 weeks of age had higher lung lesion scores in comparison with the seronegative counterparts. Nonetheless, a statistical tendency between individual S/P ratios and lung lesion scores was observed at 22 weeks of age (r = 0.49, *p* = 0.056).

**Table 3:** *M*. *hyopneumoniae* serological results from all pigs at all time points and pathological results obtained along sequential necropsies from the chronological herd study. Different superscripts within a row indicate significant differences between weeks of age (*p*<0.05). NA, non-available.

	Weeks of age							
	1	3	6	9	12	15	18	22
**No. of animals tested (n)**	58	58	58	58	48	36	29	16
**No. of seropositive animals (%)**	5 (8.6)^a^	3 (5.2)^a^	2 (3.4)^a^	3 (5.2)^a^	2 (4.2)^a^	6 (16.7)^b^	15 (51.7)^c^	11 (68.8)^c^
**Mean S/P (±SD)**	0.18 ± (0.59)^a^	0.06 ± (0.18)^a^	0.07 ± (0.23)^a^	0.08 ± (0.18)^a^	0.11 ± (0.36)^a^	0.33 ± (0.65)^b^	1.01 ± (1.35)^c^	1.46 ± (1.32)^c^
**No. of necropsied animals (n)**	NA	NA	NA	10	12	7	13	16
**No. of animals with CVPC (%)**	NA	NA	NA	0 (0)^a^	4 (33.3)^b^	6 (85.7)^c^	8 (61.5)^b, c^	13 (81.3)^c^
**Mean Ph. Eur. Score (±SD)**	NA	NA	NA	0,00 ± (0,00)^a^	1.35 ± (2.35)^b^	3.32 ± (2.57)^b^	3.09 ± (7.63)^c^	6.94 ± (8.53)^c^

#### Serum IgG1 and IgG2 subclasses

**Fig 4:**
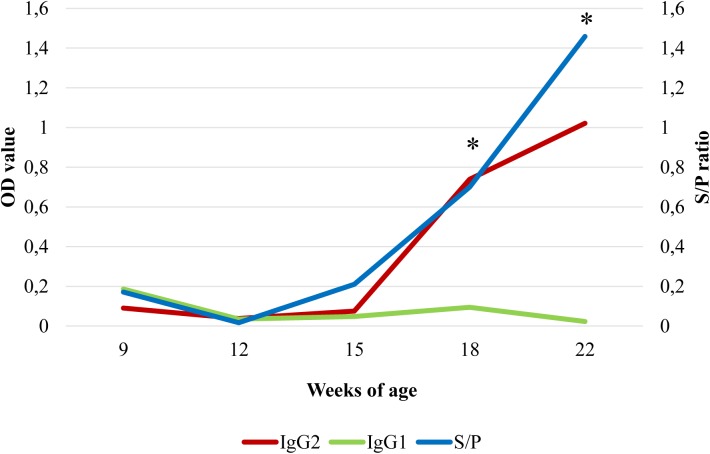
Mean S/P ratios and mean IgG1 and IgG2 OD values in sera from pigs sequentially necropsied in the chronological herd study. *Significant differences among mean IgG1 and IgG2 OD values (*p*<0.05).

Following the seroconversion pattern, mean *M*. *hyopneumoniae* IgG2 levels increased progressively from 15 weeks of age until the end of the study (22 weeks), when the mean maximum IgG2 levels in sera were reached (Fig 4). On the contrary, IgG1 mean levels maintained below the positivity threshold along the studied period but at 9 weeks of age. Indeed, mean IgG2 serum levels were significantly higher (*p*<0.05) than IgG1 ones at both 18 and 22 weeks of age. Additionally, a positive significant correlation was found between S/P ratios and IgG2 OD values in 18 (r = 0.83, *p* = 0.0004,) and 22 (r = 0.66, *p* = 0.006) week-old pigs. Notwithstanding, individual IgG2 OD values were positively correlated with mean lung lesion scores in 12 (r = 0.81, *p* = 0.001), 15 (r = 0.83, *p* = 0.04), 18 (r = 0.71, *p* = 0.063) and 22 (r = 0.62, *p* = 0.01) week-old animals.

## Discussion

This study was motivated by the interest to identify an easy *ante-mortem* parameter that would be used to reliably forecast the prevalence and severity of CVPC during the life of the pig. While serology (particularly useful for economical, rapid and high-throughput sample analysis) is the most common monitoring tool used to determine the *M*. *hyopneumoniae* status of a pig population [3], several studies have reported a positive association between serum antibody levels against *M*. *hyopneumoniae* and CVPC [2, 7–10]. On that basis, this work further studied the value of the humoral immune response to *M*. *hyopneumoniae* as a CVPC estimator by describing the association between different specific antibody isotype responses, both at local and systemic levels, and CVPC. Such putative relationship was evaluated in three different scenarios. First, experimental conditions allow modelling the acute phase of *M*. *hyopneumoniae* infection. Second, the relevance of CVPC is commonly assessed in slaughter-aged pigs [1, 2, 6], although no information about the *M*. *hyopneumoniae* infectious status is known. Lastly, pigs chronologically necropsied enabled the assessment of such relationship at different time points along *M*. *hyopneumoniae* infection dynamics. Despite CVP is frequently attributed to *M*. *hyopneumoniae* infection [1, 2, 7], it might be caused by other microorganisms (e.g. *Pasteurella multocida* and swine influenza virus) as well as influenced by farm management and housing conditions [1, 2, 7, 12, 16]. Although the present results confirm the presence of *M*. *hyopneumoniae* circulation in herds from both the slaughterhouse and chronological studies [2, 12], it should be considered that the aforesaid factors may have influenced CVPC and, in turn, the reliability of the association with the humoral immune response to *M*. *hyopneumoniae*.

At the experimental level, the local humoral immune response was stronger than the systemic one after three weeks of inoculation. Inversely, once the systemic humoral immune response began to increase by 28 dpi, revealed by a higher number of seropositive animals and increased IgG1 and IgG2 responses, a decline of both IgG and IgA antibody levels in BALF was observed. In the present work, higher levels of specific IgG and IgA mucosal antibodies were detected in those animals with CVPC in comparison with those without lung lesions. In agreement, there is a remarkable increase in the number of cells producing immunoglobulins A and G in the lungs of *M*. *hyopneumoniae* experimentally infected pigs [17–19]. Additionally, the number of cells producing specific IgG appeared to be notably higher than the ones producing IgA at lymphoid and lung tissue levels [19]. Actually, although with low to moderate correlation, specific IgG antibody amounts in BALF were found to be the best parameter to foresee CVPC at the level of the individual pig. While IgA antibodies associate with the mucosal layer in the nasal cavity, trachea and bronchi and would block *M*. *hyopneumoniae* attachment to epithelial cells, IgG antibodies are mainly found in the alveoli, being more active at lung parenchyma level through participation in opsonization and phagocytosis [20, 21]. Therefore, differential location of IgG and IgA in the respiratory tract might explain why BALF IgG levels were better correlated with CVPC scores. Similarly to earlier results [22], the present findings from experimentally inoculated pigs confirm that the humoral respiratory immune response against *M*. *hyopneumoniae* develops faster than the systemic one, which is commonly delayed in onset but persists longer. Henceforth, in an early stage of *M*. *hyopneumoniae* infection, a mucosal-based antibody measurement (such in BALF and tracheobronchial lavage samples) would be probably better as a CVPC predictor than a serological-based one.

While the local humoral immune response could play a major role in the pathogenesis of *M*. *hyopneumoniae* acute infection, IgG at the systemic level may be more important in later phases. As supporting evidence of this notion, a relationship between pneumonic lesions and IgG serum levels against *Mycoplasma pulmonis* in mice has been indicated [23]. Noticeable, reduced production of IgG was one of the most prominent characteristics in the immunological function of a mycoplasmal pneumonia resistance Landrace line [24]. Results obtained from slaughtered and chronologically necropsied pigs suggest that the amount of *M*. *hyopneumoniae* specific IgG might be related with the severity of CVPC and that could help to forecast prevalence and severity of EP-like lung lesions using a population based approach. However, the S/P value was not a good measure to predict severity of CVPC at an individual animal or farm level. Unfortunately, the specific relationship between serological responses and lung lesion scores in the 20 sampled slaughtered animals per farm was not possible. Thus, the association between both parameters at an individual slaughter weight animal level could not be assessed.

Lung lesions progress and regress throughout the lives of the pigs [6, 25, 26]. While pigs can show recovering lung lesions 8 to 12 weeks after *M*. *hyopneumoniae* exposure [27, 28], serum specific antibodies persist for longer periods [29, 30]. In fact, CVPC severity may depend on the time elapsed from *M*. *hyopneumoniae* seroconversion to the slaughter [26, 27, 31]. Thus, the main limitations to describe CVPC prevalence and severity in slaughtered pigs are due to the fact that CVPC consequence of early *M*. *hyopneumoniae* infections would probably escape detection, whereas animals infected close to the slaughter date might show CVPC but no seroconversion. For a better understanding of the humoral immunity value to forecast lifetime CVPC, samples from pigs that were followed up from birth to 22 weeks of age with sequential necropsies were evaluated. In such scenario, CVPC appeared earlier (at 12 weeks of age) than evident seroconversion (15 weeks of age) and both the percentage of seropositive pigs and animals with lung lesions increased progressively towards the end of the finishing period. Henceforth and similar to what was seen in slaughtered pigs, seropositivity was linked with more severe CVPC, but no significant correlation was observed between individual S/P ratios and lung lesion scores at any time point. While the chronological study was performed in non-vaccinated pigs, slaughtered animals belonged to batches from vaccinated and non-vaccinated herds. Although CVPC in slaughtered pigs was not importantly affected by *M*. *hyopneumoniae* vaccination [2], we cannot completely rule out that vaccination could affect the association pattern between serology and CVPC.

A novel approach aimed to determine whether there was a measurable difference in IgG1 and/or IgG2 subclasses in pig sera with and without CVPC was raised. Predominance of one IgG subclass over another in an immune response suggests Th1/Th2 bias [32]. Dominant secretion of IgG1 antibodies conveys differentiation of naïve Th cells into Th2, which are involved mainly in the activation of the humoral part of the immune response. In contrast, major detection of IgG2 is related to Th1 cells activation, which preferentially promotes the cellular immune response [32, 33]. The relevant immune response type and IgG subclass distribution could be critical for protection against a particular disease [34]. However, the involvement of IgGl and IgG2 subclass responses to *M*. *hyopneumoniae* infection and the ratio of these subclasses with respect to protection are not yet known. In the murine respiratory mycoplasma infection model, specific Th2 cell responses were found to be involved in the development of inflammatory lung lesions. In contrast, the ability to generate a Th1 cell-mediated lung immune response was related with more protection [35]. Remarkably, while this functional polarization of the Th cells has been extensively studied in mice and humans, in pigs it is less defined [33].

In the present study, the systemic humoral immune response in both experimental and natural *M*. *hyopneumoniae* infections was predominate the IgG2 subclass. In fact, IgG2 OD values and S/P values (total IgG) were found to be significantly and positively correlated. Moreover, the chronological study revealed that this antibody subclass level developed in parallel with the specific IgG response, indicating that IgG2 might probably be the main component of the systemic humoral immune response against *M*. *hyopneumoniae*. Contrarily, previous studies have reported a higher IgG1 than IgG2 antibody response in pigs challenged with *M*. *hyopneumoniae* at local [36, 37] and systemic [36] levels. Although reasons for such inconsistencies are unknown, while in such previous studies IgG1 and IgG2 subclasses against P46 [37] or P97 [36] proteins were detected, in the present case, detected antibodies were directed to a recombinant *M*. *hyopneumoniae* antigen. The latter might have had a certain influence on the observed IgG isotype bias, as it has been demonstrated that the antigen may determine the type of immune response that develops [33]. Notwithstanding, IgG2 OD values were found to be better estimators of lifetime pneumonia than S/P values. Indeed, the IgG2 OD value was the humoral immune parameter that better correlated with the lung lesion score. Despite the fact that a Th1 shifted immune response has been described in a mycoplasmal pneumonia resistant Landrace genetic line [24], present results suggest the absence of a protective role of IgG2 subclass against *M*. *hyopneumoniae*. Interestingly, Th1 immune response is required for host defense against intracellular pathogens [20, 33]. Although *M*. *hyopneumoniae* is regarded as an extracellular bacteria [38], it has been recently speculated as an intracellular pathogen [39]. Altogether, the present finding of IgG isotype bias as a correlate of *M*. *hyopneumoniae* associated lung lesions may provide an incentive for further evaluation of the Th immune response in *M*. *hyopneumoniae* infection scenarios.

## Conclusions

The present study provides insight into those potential mechanisms of immunity that might be implicated in the pathogenesis of *M*. *hyopneumoniae* infection in pigs. To our knowledge, this is the first study examining the involvement of different *M*. *hyopneumoniae* specific antibody isotype responses in lung lesion development. The results indicated an increased antibody-specific production that most likely is associated to lung lesion severity during infection. Since *M*. *hyopneumoniae* systemic humoral immune response is delayed in onset, a mucosal-based antibody measurement would be preferably chosen to predict lung lesion severity at early stages of infection. Being *M*. *hyopneumoniae* monitoring by ELISA one of the most common practices on swine farms, the obtained results may give an indication of the lung lesion severity in a certain batch. In addition, there was a clear trend towards the IgG2 response, which may reflect a dominant Th1-controlled antibody-mediated immune response. However, the measurement of other parameters related with the cellular immune response would be required to further study the role that the Th1/Th2 bias could have in *M*. *hyopneumoniae* infections.

## Supporting information

S1 Table Data bases of experimental, chronological and slaughterhouse studies. (XLSX)
